# Genetic Analysis of Membrane Cofactor Protein (CD46) of the Complement System in Women with and without Preeclamptic Pregnancies

**DOI:** 10.1371/journal.pone.0117840

**Published:** 2015-02-24

**Authors:** A. Inkeri Lokki, Tia Aalto-Viljakainen, Seppo Meri, Hannele Laivuori

**Affiliations:** 1 Department of Medical Genetics, Haartman Institute, University of Helsinki, Helsinki, Finland; 2 Department of Bacteriology and Immunology, Haartman Institute, University of Helsinki, Helsinki, Finland; 3 Immunobiology research program, Research Programs Unit, University of Helsinki, Helsinki, Finland; 4 Department of Obstetrics and Gynecology, University of Helsinki and Helsinki University Central Hospital, Helsinki, Finland; 5 Finnish Institute for Molecular Medicine, University of Helsinki, Helsinki, Finland; Queen’s University, CANADA

## Abstract

Preeclampsia is a common disorder of pregnancy characterized by endothelial dysfunction. It may be life-threatening for the mother and fetus in severe cases. Dysregulation of the complement system has been suggested to predispose women to preeclampsia. Complement is part of the innate and adaptive immune systems and potentially capable of causing inflammation and tissue damage. Membrane cofactor protein MCP (CD46) is among the potent complement regulators that have recently been linked to a severe form of preeclampsia with or without an underlying autoimmune phenotype. Mutations in *CD46* predispose to thrombotic microangiopathy with endothelial cell dysfunction. The exome of *CD46* were sequenced in 95 Finnish women with severe preeclampsia. Genetic variations discovered in the full exome were compared to those observed in 95 control women who did not develop preeclampsia. Because A304V (rs35366573) was associated with preeclampsia in one previous study, we sequenced the transmembrane region including the A304V variant and part of the cytoplasmic tail in 95 additional controls. We did not discover any association between A304V or other *CD46* SNPs and preeclampsia. This study describes a carefully characterized cohort of severely preeclamptic Finnish women and found no potentially predisposing variants in *CD46*. However, it is possible that other genetic components of the complement system may affect the pathogenesis of severe preeclampsia and related diseases.

## Introduction

Preeclampsia is a common (3–5%) disorder of pregnancy. It increases perinatal mortality five-fold [1]. The onset and clinical course of preeclampsia is unpredictable. It often necessitates preterm delivery, which carries the risk for complications of prematurity. Preeclampsia is also associated with an increased risk of cardiovascular disease in later life of both the mother and the fetus [2,3]. Although the disease originates early in pregnancy, the defining symptoms, newly-onset hypertension and proteinuria, develop in the latter half of pregnancy. Currently, no reliable predictive test for preeclampsia is known and no cure other than delivery is available.

**Fig 1:**
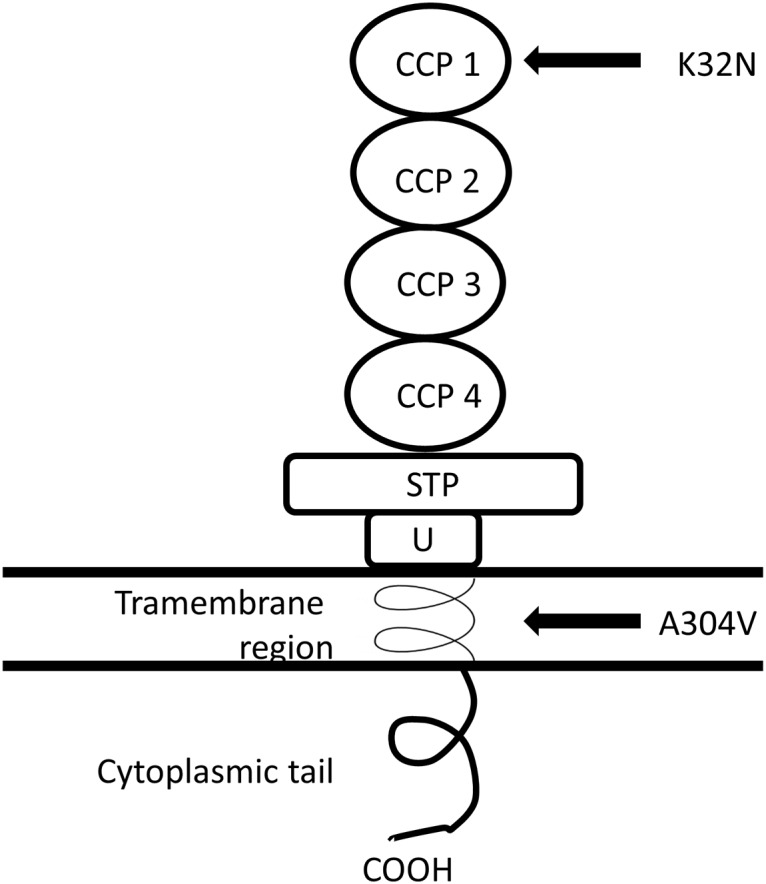
The schematic structure of the membrane cofactor protein (CD46). The positions of the amino acid changing single nucleotide polymorphisms described in Salmon *et al*. [12] and in this study are depicted. Inspired by Fang *et al*. [22].

The complement system is an integral part of the innate immune system consisting of phylogenetically ancient processes of pathogen recognition and self-/non-self-discrimination. In a normal pregnancy the complement system becomes activated at a low level [4]. Activation of the terminal complement pathway leads to formation of the membrane attack complex (MAC) and possible destruction of the target cell. Complement can be activated via three independent pathways. The lectin pathway (LP) is activated by mannose-binding lectin (MBL) and by ficolins. It has previously been studied in association with preeclampsia with contradictory results [4,5]. The classical pathway (CP) is activated by the C-reactive protein (CRP) or by immune complexes, such as antibodies bound to microbes or autoantibodies bound to self-antigens. The role of a microbial trigger for inflammation during pregnancy as a causative agent in preeclampsia has been the subject of a lengthy debate, where no definitive conclusions have yet been achieved [6–9]. An important role for the alternative pathway (AP) of complement in abnormal pregnancy was suggested by animal studies over a decade ago, when Crry, the functional murine homologue of human complement inhibitors membrane cofactor protein (MCP) and decay accelerating factor (DAF), was knocked out causing infertility due to alternative pathway activation [10]. Recently, it was shown that an elevated level of alternative pathway activation occurs also in human pregnancies, where severe preeclampsia develops [11]. Activation of the complement system must be rigorously regulated in order for normal placentation to occur and for the placenta to be protected from tissue destruction, thrombosis and antiangiogenic factors [12,13]. MCP is a widely expressed complement regulator that inhibits AP, LP and CP. It binds to both C3b (AP) and C4b (CP) and acts as a cofactor for their inactivation by the C3b/C4b inactivator enzyme factor I [14,15]. MCP is a type 1 membrane bound protein consisting of an intracellular tail region with several variant structures [7] a single transmembrane α-helical region, a short region of unknown function (U), a collar STP- rich region with several variant structures, and finally four extracellular Complement Control Protein (CCP) domains carrying the cofactor activity (Fig. 1) [15,16].

MCP is encoded by *CD46* located in the Regulators of Complement Activation gene cluster on chromosome 1q32.2. Apart from its role as a complement inhibitor on cell membranes MCP can act as a receptor for certain pathogens such as the measles virus (Edmonston strain) [17], human herpesvirus 6 [18] and *Neisseria gonorrhoeae* [19]. MCP has also been suggested to have role in the fertilization of the human egg [20]. Notably, several mutations both in the CCPs as well as in the transmembrane region are linked to atypical hemolytic uremic syndrome (aHUS) [21,22].While we initiated our studies on the AP regulatory genes in preeclampsia, MCP among other complement regulators received considerable attention as potential target for mutations in preeclampsia [12].

We hypothesized that variation in complement regulation might be one underlying reason for severe preeclampsia in patients who developed heavy proteinuria. The objective of this study was to investigate whether sequence variations in the *CD46* promoter, splice sites and exome are present in Finnish women with and without preeclampsia. Because A304V (rs35366573) was associated with preeclampsia in one previous study [12], we studied this variant in a larger group of controls.

## Methods

### STUDY POPULATION

The participants are a subgroup of the FINNPEC (The Finnish Genetics of Preeclampsia Consortium) study cohort. Briefly, at the time of publication, the cohort comprises 1090 women diagnosed with preeclampsia in their singleton pregnancy and 930 women without preeclampsia symptoms in their singleton pregnancy (the control group). Preeclampsia was defined as hypertension and proteinuria occurring after 20 week of gestation. Hypertension was defined as a systolic blood pressure ≥ 140 mmHg, and/or a diastolic blood pressure ≥ 90 mmHg. Proteinuria was defined as the urinary excretion of ≥0.3 g protein in a 24-hour specimen, or 0.3g/L, or ≥1+ reading on dipstick in a random urine determination at least twice with no evidence of a urinary tract infection. Intrauterine growth restriction (IUGR) was defined as birth weight below-2 SD according to the Finnish standards [23].

For this study we selected a subset of 95 primiparous women with severe preeclampsia (a systolic blood pressure ≥160 mmHg or a diastolic blood pressure ≥110 mmHg or proteinuria ≥5 g protein in a 24-hour specimen). Because the prominent mutations of *CD46* are known to associate with disorders involving kidney function, we included preeclamptic women with heavy proteinuria in this study. They had no autoimmune diseases. Twenty-six patients (27%) had early-onset preeclampsia (diagnosis before 34 weeks of gestation). A control group comprised 190 women randomly selected from the original control group. None of the controls had autoimmune diseases. The clinical characteristics of the two groups are shown in Table 1.

**Table 1:** Clinical characteristics of the study population.

	Severe preeclampsia n = 95	Controls n = 190	Difference between patients and controls
	Mean (SD) N (%)	Mean (SD) N (%)	
Age (years)	29.0 (4.55)	30.8 (4.87)	p = 0.003^†^
Body mass index (kg)	24.2 (4.92)	23.9 (3.72)	NS^†^
Primipara	95 (100%)	104 (55%)	p < 0.001^‡^
Early-onset preeclampsia (diagnoses <34 weeks of gestation)	26 (27%)	0	p < 0.001^‡^
Systolic blood pressure (mmHg)	173 (15.36)	125 (12.94)	p < 0.001^†^
Diastolic blood pressure (mmHg)	112 (6.92)	83 (7.99)	p < 0.001^†^
Proteinuria (g, 24-hour specimen)	5.8 (4.49)	*	
Gestational diabetes	2 (2%)	10 (5%)	p = 0.005^‡^
Pre-gestational diabetes (type 1 diabetes)	0	3 (2%)	NS^‡^
Pre-gestational hypertension	1 (1%)	3 (2%)	NS^‡^
Gestational hypertension	0	7 (4%)	p = 0.043^‡^
Weeks of gestation at delivery	36.2 (2.89)	39.9 (1.58)	p < 0.001^§^ ^†^
Preterm birth (delivery before 37 weeks of gestation)	39 (42%)	6 (3.2%)	p < 0.001^‡^
Birthweight (g)	2582 (733.86)	3622 (474.27)	p < 0.001^§^ ^†^
Intrauterine growth restriction (<-2 SD)	18 (19%)	1 (<1%)	p < 0.001^‡^

The 95 subgrou of controls did not differ statistically from the 190 control pool. The significance level is 0.05.

NS—not significant

^§^ Equal variances not assumed

* One individual (0.5%) in the control group had gestational proteinuria.

^†^ T-test

^‡^ Mann-Whitney U test

### ETHICS STATEMENT

All subjects provided a written informed consent and the FINNPEC study protocol was approved by the coordinating Ethics Committee of the Hospital District of Helsinki and Uusimaa (permit number 149/E0/07).

### LABORATORY METHODS

DNA was extracted from 10 ml EDTA whole blood stored at-20C° (after initial freezing period at-80C° to prevent formation of icicles and molecular degradation) using Chemagic Magnetic Separation Module I (Chemagen, PerkinElmer, Baesweiler, Germany) automatic DNA extraction protocol as provided by the manufacturer. Extracted DNA was used at final concentrations of 20 ng /μl and 30 ng /μl.

Exomic sequencing inclusive of flanking intronic regions was carried out using 15 pairs of primers as listed in S1 Table. Promoter region 240 bp upstream of exon 1 was also sequenced. Primers were designed and tested by using Primer3 and GenomeTester softwares [24,25]. PCR was carried out using Mytaq (Bioline, London, UK) polymerase enzyme and buffer following the manufacturer’s protocol. PCR products were purified using ExoSAP-IT (GE Healthcare Life Science, UK) shrimp phosphatase alkaline product and the purified DNA fragments were sequenced by Big Dye Terminator v3.1 Cycle enzyme and buffer (Applied Biosystems, Carlsbad, CA, USA) following the manufacturer’s protocol. The sequencing reaction product was purified by Performa DTR v3 filterplates (Edge BioSystems, Gaithersburg, MD, USA) and analyzed using ABI3730xl capillary electrophoresis sequencer (Applied Biosystems, Carlsbad, CA, USA).

The raw data was analyzed using Sequencher 8 software (Applied Biosystems, Carlsbad, CA, USA) and the detected polymorphisms were verified using SNPper [26] and NCBI databases.

### STATISTICAL METHODS

*CD46* sequencing results were analyzed for disease association in PLINK [27]. Association by individual SNPs and association in terms of different genotypic models were evaluated by Fisher’s Exact test. The functionality of amino acid changing SNPs was evaluated using PolyPhen-2 [28] and SIFT [29]. In SIFT, scores < 0.05 or sometimes < 0.1 are considered indication of a deleterious mutation [30]. An independent values t-test was used to compare means of clinical characteristics between patients and controls in IBM SPSS statistics version 22 (IBM corp.).

## Results

**Fig 2:**
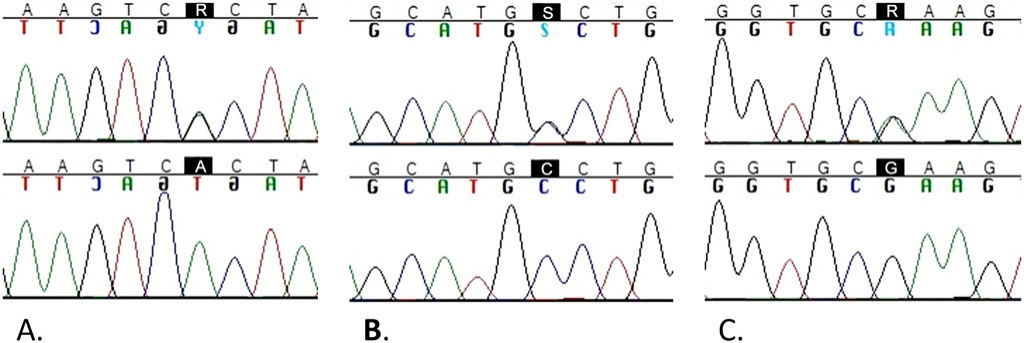
The sequences of the newly discovered SNPs near exon 13 and in exon 14 of the *CD46*. Panel A: New SNP1 G/A heterozygote (top) and A/A homozygote (bottom). Panel B: New SNP2 G/C heterozygote (top) and C/C homozygote (bottom). Panel C: New SNP3 A/G heterozygote (top) and G/G homozygote (bottom).

A total of 15 SNPs (Table 2), including three novel ones (Table 3), were observed in *CD46*. The alleles and minor allele frequencies (MAF) in patients and controls are presented in Table 2. Heterozygosity for the A304V variant (rs35366573) was observed in 12% (11/95) of cases and 11% (21/188) of controls (OR = 0.943, 95% CI = 0.450, 1.978). In addition, one control was homozygous for A304V. A304V was predicted to be benign (Polyphen2 score 0.011/1) and tolerated (Sift: 0.19). The K32N variant (rs150429980), an amino acid change in the functional part of the MCP molecule, in the most membrane distal CCP, was observed in a heterozygous form in one patient and in one control (OR = 1, 95% CI = 0.062, 16.11, S2 Table). K32N was ambivalently predicted to be probably damaging (Polyphen2 score 0.996/1, sensitivity 0.55, specificity 0.98) or possibly tolerated (Sift: 0.13). One new SNP was located near exon 13, being observed in one control. Two of the new SNPs were located in exon 14. Both were observed once in a different patient (Fig. 2, Table 3).

**Table 2:** The observed single nucleotide polymorphisms (SNPs) and minor allele frequencies (MAF) in the genotyped cases and controls.

CHR	SNP	MAF cases	MAF controls	N cases	N controls	Location
1	rs2796268	0.453	0.457	95	94	promoter
1	rs41266397	0.069	0.074	95	95	promoter
1	rs150429980	0.005	0.005	95	94	exon 2 K32N
1	rs12126088	0.011	0.000	95	94	exon 4 synonymous
1	rs41258244	0.069	0.074	95	94	exon 5 synonymous
1	rs2724374	0.247	0.200	95	95	intronic
1	rs35366573	0.058	0.061	95	188	exon 11 A304V
1	NewSNP1	0.000	0.005	92	97	intronic
1	NewSNP2	0.005	0.000	95	94	cytoplasmic tail
1	NewSNP3	0.005	0.000	95	187	cytoplasmic tail
1	rs7144	0.452	0.457	95	94	cytoplasmic tail
1	rs193023975	0.005	0.000	95	94	cytoplasmic tail
1	rs185457983	0.000	0.005	95	94	cytoplasmic tail
1	rs14374	0.053	0.021	95	95	cytoplasmic tail
1	rs1237	0.069	0.042	95	95	cytoplasmic tail

**Table 3:** Details of the new single nucleotide polymorphisms (SNPs).

SNP ID	Allele 1	Allele 2	Genomic position*
NewSNP1	G	A	207963561
NewSNP2	G	C	207967178
NewSNP3	A	G	207967697

* according to build GRCh37.p10

## Discussion

In this study we did not find any sequence variants in promoters, exomes and flanking regions of *CD46* associated with preeclampsia. Preeclampsia is known to be a clinically variable and etiologically heterogeneous disease [31]. Preeclamptic patients with heavy proteinuria were selected for the project, because several previous studies pointed towards functional problems of the kidney in association with complement activation and particularly with *CD46* mutations, as observed in aHUS [17,21,22,32]. The similar nephrological symptoms between aHUS and preeclampsia led us to pursue a common cause to these two conditions.

Interestingly, Salmon and co-workers reported *CD46* mutations as genetic defects associated with preeclampsia [12]. In the PROMISSE cohort consisting of 250 pregnant patients with *systemic lupus erythematosus (SLE)* and/or antiphospholipid antibody (*APL Ab*) syndrome they found an increase from 2.5% to 7% in A304V heterozygosity in women, who developed preeclampsia. They replicated their finding in 59 women with preeclampsia and/or the *HELLP* (hemolysis, elevated liver enzymes, low platelet count) syndrome and 143 controls without autoimmune diasease [12]. In the 59 patients, Salmon *et al*. observed a higher MAF (minor allele frequency 3.4%) than what was seen in ethnically and geographically matched controls (1.4%, including 1 homozygous individual) [12]. Our results with a larger study population do not support the previously reported findings. In the present study, A304V variant was equally prevalent in the two groups. Furthermore, we discovered an individual homozygous for A304V among the healthy controls. MAF for A304V was 5.4% in 3316 Finnish individuals of the SISu (Sequencing initiative Suomi) data resource (www.sisuproject.fi). While NCBI reports heterozygosity in different populations to range between 2–4%, with MAF of 0.6–2.2%, the highest values being recorded in European populations, our result together with the SISu data suggests, that an even higher MAF is typical of the general Finnish population. In light of this evidence it seems unlikely that the increase in MAF from 2.1% (general population) or 1.4% (healthy geo-ethnically matched pregnant controls) to 3.7% (PROMISSE with preeclampsia) or 3.4% (Utah Severe preeclampsia/HELLP cohort) alone would bear functional consequences for the development or progression of preeclampsia, as previously suggested [12]. Furthermore, preeclampsia being a heterogenous disease, different subtypes such as HELLP may not be compatible in etiological comparison. Our data set had no patients with severe immunological diagnoses such as SLE. The patients with severe preeclampsia from the study by Salmon *et al*. are comparable to our data set [12].

A304V variant is located in the transmembrane region of the molecule (Fig. 1). The possible functional role is thus not directly related to interaction with C3b and C4b, which are known to bind to extracellular CCP regions. The mechanism of decreased alternative pathway regulation associated with A304V is thus not clear [22,33]. We did not find indication of disease relatedness of A304V in our bioinformatics analyses in a majority of the transcripts, i.e. splice variants included in the software analyses.

Salmon et al. found K32N in a patient affected by SLE with a history of repeated pregnancy loss, fetal death and preeclampsia. This amino acid substitution from basic lysine to neutral asparagine causes a 4x decrease in the C4b cleavage promoting activity of MCP [12]. In our material K32N was observed in a heterozygous form in one preeclamptic patient and one control. SISu reports the MAF of K32N to be 0.03% in 3325 Finnish individuals. In the bioinformatics analyses the possible functional effects of K32N were controversial. While SIFT predicted K32N to be tolerated, Polyphen2 predicted it to have deleterious changes for the protein function in all available transcripts.

Preeclampsia being a clinically variable and heterogeneous disease, it is always a challenge to pinpoint an association to link single gene variant to a particular subphenotype. The missing association between *CD46* mutations and preeclampsia may also be attributed to incomplete penetrance of complement mutations in preeclampsia. Furthermore, the multifactorial background is another likely explanation for missing association in a complex disease like preeclampsia. The present study does not account for the effect of disease specific isoforms in preeclampsia. While several splicing isoforms of *CD46* have been known to exist for a long time recent work suggests a disease specific role for the occurrence of *CD46* isoforms [7]. Exons 13 and 14 code for the large cytoplasmic tail section of the molecule, which is variably included or spliced out from the functional isoforms of the protein (Fig. 1) [21]. The functional effects of the new SNPs discovered in these exons fundamentally depend on whether they are included in the isoform in question.

As discussed above, the complement system has an essential role in pregnancy. The overriding hypothesis is that excessive and uncontrolled activation of the maternal complement system compromises the placenta and ultimately the fetus [12,13]. Accordingly, it is not likely that malfunctioning maternal MCP, being a cell-bound regulator of the complement system, would have a fundamental effect on the integrity of tissues of fetal origin such as the placenta. Maternal activators and fetal regulators are the more obvious molecules of interest in future studies.

In this study we have explored the genetic polymorphism of *CD46* in preeclampsia. Three novel rare variants were discovered but neither they nor other polymorphisms of interest were associated with severe preeclampsia. Studies on functional *CD46* isoforms in preeclampsia may provide further insight into the possible role of MCP and complement-mediated injury in the pathogenesis of severe preeclampsia.

## Supporting Information

S1 Table Used primers. (DOCX)

S2 Table Association as determined by Fisher’s exact. (DOCX)
