# Correction: Genetic Analysis of Membrane Cofactor Protein (CD46) of the Complement System in Women with and without Preeclamptic Pregnancies

**DOI:** 10.1371/journal.pone.0125449

**Published:** 2015-04-10

**Authors:** The PLOS ONE Staff

There is an error in the author byline. Dr. Hannele Laivuori acted on behalf of FINNPEC. The correct byline is: A. Inkeri Lokki, Tia Aalto-Viljakainen, Seppo Meri, Hannele Laivuori on behalf of FINNPEC. The expanded group author list can be found in the Acknowledgements section within the manuscript. The publisher apologizes for this error.
