# Supplementary material for: Genetic Analysis of Membrane Cofactor Protein (CD46) of the Complement System in Women with and without Preeclamptic Pregnancies
Source: PLoS One. 2015 Feb 24;10(2):e0117840. doi: 10.1371/journal.pone.0117840 (PMC4339547; doi:10.1371/journal.pone.0117840)
Supplement: S1 Table — (DOCX) [file pone.0117840.s001.docx]

Supplementary table S1. Used primers and the size of amplicons.

| Region | Left primer | Right primer | Amplicon Size |
| --- | --- | --- | --- |
| promoter | CCGAATTCCCGGAAACTATT | CCGGAGAAGGAGTACAGCAG | 561 |
| exon 1 | TCGGTTTCTCTGCTTTCCTC | AGAGAACCCTGTCCCCAAAC | 265 |
| exons 2-3 | TTCCCAAACAAACCAAAAGC | CCCTTATTTCCTCTAAGGAGCA | 877 |
| exon 4 | GTGGAAAGGCACAGCAGATT | GGGTGTAAAGGAGGCAAAAA | 398 |
| exon 5 | TTGACAAATTTATTGAAGACACAGAA | CAGGAGGAGGAAGCACATACA | 392 |
| exon 6 | TTGCATTCCATTCCTTGTCTC | TCTAAAATGAACAGCAACAACAA | 348 |
| exons 7-8 | AACTCCCAAGTGGTTGATCTTC | CAAATGTCCTCCCTCCTTTC | 385 |
| exon 9 | TTGATAAGGCCCTGGTGAAT | CACGCTGTGCACACATACC | 180 |
| exon 10 | AAGGGATTTTCTACAAAGGTGAA | TGTTTGGGCACCTCATAAAA | 240 |
| exons 11-12 | TCTGGAGATCCATGTGTTCAA | ACTGAAGCTGCACAAAAGCA | 730 |
| exon 13 | TGCTACTCGTTTCTTTTTGGTTT | AGCAAAGGGAACAGGAATGT | 299 |
| exon 14.1 | CCAGGTTGGTGGCTCATTAC | TTTTTATGCACAAGAGCCAAA | 679 |
| exon 14.2 | TCTTTGTAAAGAAAGTGGCTTGAA | GCCAAGGCAATGTAAATGGA | 658 |
| exon 14.3 | AGTCTTGTTGTTTTCCCAAAGA | GAATTCCTGTTTGTTCTCCTCAA | 694 |
| exon 14.4 | TTGGGCCAAAGAAACATTG | GATGGGCCCAATTAGAAACA | 700 |
