# Supplementary material for: Genetic Analysis of Membrane Cofactor Protein (CD46) of the Complement System in Women with and without Preeclamptic Pregnancies
Source: PLoS One. 2015 Feb 24;10(2):e0117840. doi: 10.1371/journal.pone.0117840 (PMC4339547; doi:10.1371/journal.pone.0117840)
Supplement: S2 Table — (DOCX) [file pone.0117840.s002.docx]

Supplementary table S2. Association as determined by Fisher’s exact.

| CHR | SNP | BP | Allele 1 | Allele 2 | P- value | OR | SE | L95 | U95 |
| --- | --- | --- | --- | --- | --- | --- | --- | --- | --- |
| 1 | rs2796268 | 1442971 | G | A | 1 | 0.981 | 0.207 | 0.654 | 1.470 |
| 1 | rs41266397 | 1443140 | G | C | 1 | 0.923 | 0.400 | 0.422 | 2.021 |
| 1 | rs150429980 | 1448238 | T | A | 1 | 0.999 | 1.418 | 0.061 | 15.940 |
| 1 | rs12126088 | 1450790 | G | A | 0.499 | NA | NA | NA | NA |
| 1 | rs41258244 | 1452332 | T | A | 0.844 | 0.913 | 0.400 | 0.417 | 1.998 |
| 1 | rs2724374 | 1458970 | C | A | 0.325 | 1.315 | 0.247 | 0.810 | 2.135 |
| 1 | rs35366573 | 1476225 | T | C | 1 | 0.943 | 0.378 | 0.450 | 1.978 |
| 1 | NewSNP1 | 1481340 | G | A | 1 | 0 | inf | 0 | nan |
| 1 | NewSNP2 | 1484921 | G | C | 1 | NA | NA | NA | NA |
| 1 | NewSNP3 | 1485476 | A | G | 0.337 | NA | NA | NA | NA |
| 1 | rs7144 | 1485498 | C | T | 1 | 0.981 | 0.207 | 0.654 | 1.470 |
| 1 | rs193023975 | 1485712 | T | C | 1 | NA | NA | NA | NA |
| 1 | rs185457983 | 1485714 | G | A | 0.497 | 0 | inf | 0 | nan |
| 1 | rs14374 | 1486036 | C | T | 0.171 | 2.583 | 0.601 | 0.796 | 8.386 |
| 1 | rs1237 | 1486476 | G | T | 0.370 | 1.671 | 0.462 | 0.677 | 4.129 |

CHR – Chromosome, SNP – Single nucleotide polymorphism identifier, BP – Base pair, CHISQ – chi^2^ test value, OR – Odds ratio, SE – Standard error, L95 – Lower 95% confidence interval, U95 – Upper 95% confidence interval.
